# Exposure to exogenous egg cortisol does not rescue juvenile Chinook salmon body size, condition, or survival from the effects of elevated water temperatures

**DOI:** 10.1002/ece3.6073

**Published:** 2020-02-08

**Authors:** Theresa R. Warriner, Christina A. D. Semeniuk, Trevor E. Pitcher, Oliver P. Love

**Affiliations:** ^1^ Great Lakes Institute for Environmental Research University of Windsor Windsor Ontario Canada; ^2^ Department of Integrative Biology University of Windsor Windsor Ontario Canada

**Keywords:** climate change, cortisol, environmental match, growth, maternal stress, Pacific salmon, performance, prenatal stress, thermal stress

## Abstract

Climate change is leading to altered temperature regimes which are impacting aquatic life, particularly for ectothermic fish. The impacts of environmental stress can be translated across generations through maternally derived glucocorticoids, leading to altered offspring phenotypes. Although these maternal stress effects are often considered negative, recent studies suggest this maternal stress signal may prepare offspring for a similarly stressful environment (environmental match). We applied the environmental match hypothesis to examine whether a prenatal stress signal can dampen the effects of elevated water temperatures on body size, condition, and survival during early development in Chinook salmon *Oncorhynchus tshawytscha* from Lake Ontario, Canada. We exposed fertilized eggs to prenatal exogenous egg cortisol (1,000 ng/ml cortisol or 0 ng/ml control) and then reared these dosed groups at temperatures indicative of current (+0°C) and future (+3°C) temperature conditions. Offspring reared in elevated temperatures were smaller and had a lower survival at the hatchling developmental stage. Overall, we found that our exogenous cortisol dose did not dampen effects of elevated rearing temperatures (environmental match) on body size or early survival. Instead, our eyed stage survival indicates that our prenatal cortisol dose may be detrimental, as cortisol‐dosed offspring raised in elevated temperatures had lower survival than cortisol‐dosed and control reared in current temperatures. Our results suggest that a maternal stress signal may not be able to ameliorate the effects of thermal stress during early development. However, we highlight the importance of interpreting the fitness impacts of maternal stress within an environmentally relevant context.

## Graphical abstract

We aimed to test whether prenatal stress could dampen the effects of elevated water temperatures during Chinook salmon (*Oncorhynchus tshawytscha*) early development. We mimicked maternal stress by exposing fertilized eggs to 1,000 ng/ml of cortisol and incubated the eggs in current (+0°C) or elevated (+3°C) temperature regimes. As predicted, we found that elevated water temperatures resulted in smaller body size and lower survival, but our prenatal stress treatment did not ameliorate the effects of elevated temperatures on body size or survival.

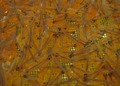

## 1 INTRODUCTION

Climate change is altering habitats across the globe, introducing environmental stressors such as elevated mean temperatures (Stocker et al., 2013), increased frequency of extreme weather events (e.g., floods, droughts; Easterling et al., 2000; Fischer & Knutti, 2015), and novel competitor and predator interactions (e.g., via species range expansions toward the poles; Parmesan & Yohe, 2003). Climate change is occurring at an accelerated rate (Loarie et al., 2009) and is consequently affecting the capacity of organisms to respond adaptively (Palmer et al., 2017; Woodward et al., 2016). Organisms may respond to environmental stressors through phenotypic changes in their growth, morphology, reproduction, and survival (Barton, 2002), which can include rapid‐acting mechanisms within and across generations such as phenotypically flexible responses, phenotypic plasticity, and contemporary evolution (Hendry, Farrugia, & Kinnison, 2008; Sih, Ferrari, & Harris, 2011). Climate change is expected to have strong direct and indirect effects on aquatic systems, with alterations in the hydrological cycle (e.g., changes in precipitation) leading to extremes in water availability (i.e., floods and droughts), increases in air temperature leading to increased water temperatures, and increased CO_2_ leading to increasing water acidity (Bates, Kundzewicz, Wu, & Palutikof, 2008). Notably, increasing temperatures alone are expected to greatly impact ectothermic organisms such as invertebrates and fishes, and oftentimes are considered to be negative (Ficke, Myrick, & Hansen, 2007; Stoks, Geerts, & De Meester, 2014). Indeed, warming waters generate offspring phenotypic traits such as smaller body size (Kuehne, Olden, & Duda, 2012; Whitney, Hinch, Patterson, & a., 2014), faster growth (Beacham & Murray, 1990), and increased metabolism (Enders & Boisclair, 2016). In turn, offspring performance and life history traits can also be affected, including lower thermal tolerance (Chen et al., 2013), earlier development (Fuhrman, Larsen, Steel, Young, & Beckman, 2018), altered migration timing (Crozier, 2015), and modified reproduction (Pankhurst & Munday, 2011), ultimately resulting in changes to fitness (e.g., survival; Martins et al., 2012; Whitney, Hinch, & Patterson, 2013). Examining the underlying phenotypic plasticity mediating these effects is important to determine how we expect species to fare under future climate scenarios (Hoffmann & Sgró, 2011; Merilä & Hendry, 2014). Yet, we still know fairly little about the mechanism(s) inducing plasticity (Monaghan, 2008), and whether temperature effects can be further altered by additional environmental modulators (i.e., maternal effects; Galloway, 2005).

Some potent modulators of adaptive phenotypic responses in offspring include maternal effects (Green, 2008; Meylan, Miles, & Clobert, 2012) or epigenetic programming (Bonduriansky, Crean, & Day, 2012). Maternal effects have long been recognized for the role in the fine‐scale tuning of adaptive responses to larger environmental stressors (Räsänen & Kruuk, 2007). For example, maternal exposure to environmental stressors translates information to developing offspring about the relative quality of their future environment (Sheriff et al., 2017). Maternally derived hormones have recently been acknowledged as a possible mechanism by which information about environmental quality can be translated to offspring via the mother (Meylan et al., 2012). In particular, maternally derived glucocorticoid (GC) hormones have been proposed as preparative mediators of offspring phenotype and performance in relation to the predicted quality of the offspring's future environment (Love, Chin, Wynne‐Edwards, & Williams, 2005; Sheriff & Love, 2013). Glucocorticoids are strong candidates for this mediatory effect since they are involved in energy management and the stress response in vertebrates (Barton, 2002; Moore & Jessop, 2003; Romero, 2004). As such, environmental stressors can elevate maternal baseline GCs (Love, McGowan, & Sheriff, 2013; Wendelaar Bonga, 1997), and maternally derived GCs can be transferred to developing offspring in utero (Matthews, 2002) or via the lipid content of eggs in oviparous species (Love et al., 2009; Sopinka, Capelle, Semeniuk, & Love, 2017) providing a reliable signal of current and potentially future environmental quality for offspring (Love et al., 2013). Recent studies suggest that when the maternal environment is indicative of the offspring environment (environmental match hypothesis; Sheriff et al., 2017; Sheriff & Love, 2013), maternal stress may elicit predictive adaptive responses (PARs) in offspring (Bateson, Gluckman, & Hanson, 2014), generating offspring phenotypes that may be better prepared for harsher environments (Bian et al., 2015; Gagliano & McCormick, 2009; Love et al., 2013; Love & Williams, 2008). From a climate change perspective, where the projected global mean surface temperatures are expected to increase 0.3–0.7°C by 2035 (Stocker et al., 2013) and where harsher events such as extreme water flow (i.e., flood and droughts) are expected to be more frequent (Woodward et al., 2016), females may be exposed to multiple environmental stressors during reproduction. Thus, maternally derived GCs may be involved in modulating the responses of ectothermic offspring to multiple environmental stressors due to climate change, by better‐preparing offspring for managing harsher environmental conditions such as elevated temperatures during development (Sopinka et al., 2017).

**Figure 1:**
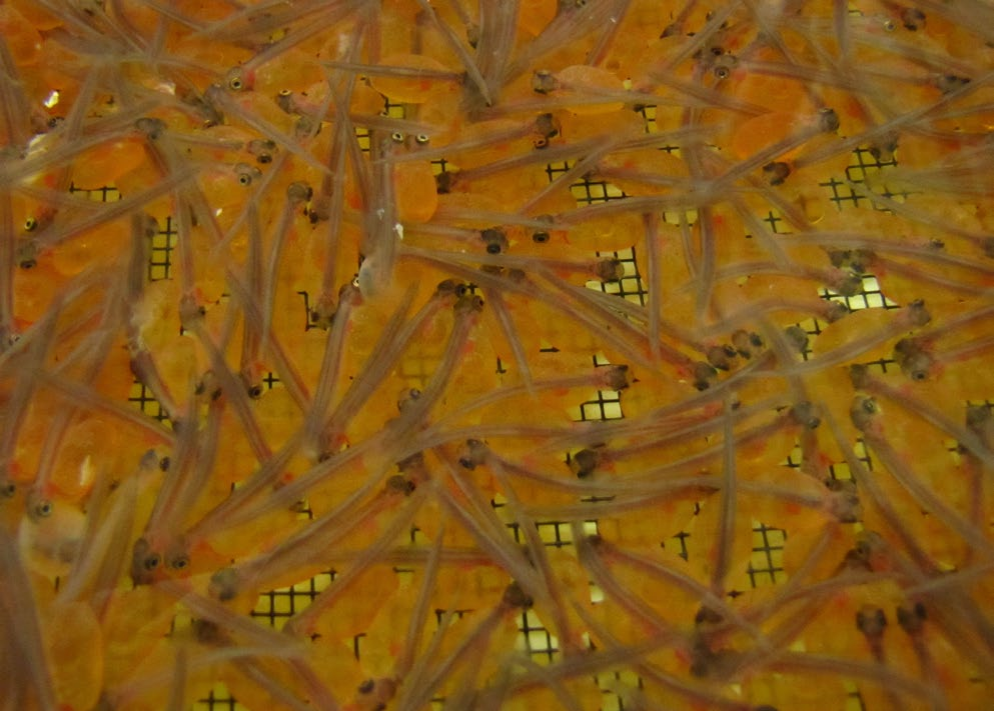
Picture of Chinook salmon (*Oncorhynchus tshawytscha*) hatchlings. Photo Credit: T. Warriner

Here, we apply the concept of environmental matching (Sheriff & Love, 2013) to examine whether exposure to maternally derived GCs can generate phenotypes that better buffer the negative phenotypic effects of developing in elevated water temperatures using Chinook salmon *Oncorhynchus tshawytscha* (Figure 1). Pacific salmon are an important study species for these questions since they are ectothermic and are sensitive to changes in environmental temperatures (McCullough et al., 2009); are susceptible to additional stressors during migration (i.e., increase circulating plasma GCs) when they return to terminally spawn (Cook et al., 2014; McConnachie et al., 2012); and mothers and offspring overlap spatially in their spawning and developing environment, respectively, meaning that an honest signal about environmental quality passed from mother to eggs may be valuable for salmon offspring. Importantly, climate change has been implicated for declines in Chinook salmon populations across the west coast of North America, through direct and indirect effects of water temperature increases and droughts (Isaak, Wollrab, Horan, & Chandler, 2012; Mantua, Tohver, & Hamlet, 2010). Determining whether offspring are better prepared for warmer waters after having received a hormonal maternal signal about increased environmental stress is an important component for quantifying the adaptive capacity of Chinook salmon to climate change. To investigate how the effects of elevated temperatures and maternally derived GCs interact to affect Chinook offspring phenotype and performance, we exposed eggs to exogenous cortisol or a control solution immediately postfertilization and then split these eggs within females and raised the offspring in one of two temperature regimes: current (+0°C) and elevated (+3°C: as predicted in next century by current climate models, Vliet, Franssen, et al., 2013). In accordance with previous research (e.g., Daufresne, Lengfellner, & Sommer, 2009; Whitney et al., 2014), we predicted that individuals raised in elevated temperatures would have both lower survival and body size than those raised in current temperatures. However, since preparatory responses following exposure to a maternal stress signal may dampen the effects of an environmental stressor (i.e., environmental matching hypothesis; Sheriff et al., 2017), we predicted that prenatal exposure to exogenous cortisol would help to buffer these negative impacts, resulting in relative increases in survival and body size at emergence (fry stage) for fish raised under elevated water temperatures.

## 2 METHODS

### 2.1 Fish Origin

All work described here was approved and completed under University of Windsor Animal Use Project Proposals (AUPPs: # 14‐25 & #15‐15). Our study species was Chinook salmon from the Credit River (43°34′40.0″N 79°42′06.3″W), which drains into Lake Ontario, Canada. Chinook salmon were purposely introduced to Lake Ontario starting in 1967 and they continue to be stocked for recreational fishing purposes (OMNRF, 2015). Spawning in this Great Lake's population takes place in the early fall, where eggs incubate under gravel in these natal streams until hatching in February and emergence in March, with juveniles migrating out into the lake in summer (Johnson, 2014). Tributaries that flow into the Great Lakes are expected to increase in water temperature (Chu, 2015; Vliet, Franssen, et al., 2013), and therefore investigating this population's responses to these environmental changes may provide information relevant to the future status of Great Lakes fisheries under climate change. We collected eggs and milt from fifteen adult females and nine males on October 4, 2016, from the Credit River. We measured female body mass (mean ± SE, range: 7.89 ± 0.41, 5.5–11.7 kg), fork length (85.6 ± 0.89, 80.0–93.0 cm), and ovarian mass (ovarian mass = pre–post stripping mass: 0.98 kg, ±0.10, 0.55–2.00 kg). Eggs and milt were collected by applying pressure to the abdomen, and the gametes were transported on ice in coolers to the University of Windsor's Aquatic Facility.

### 2.2 Egg cortisol exposure and incubation temperatures

**Figure 2:**
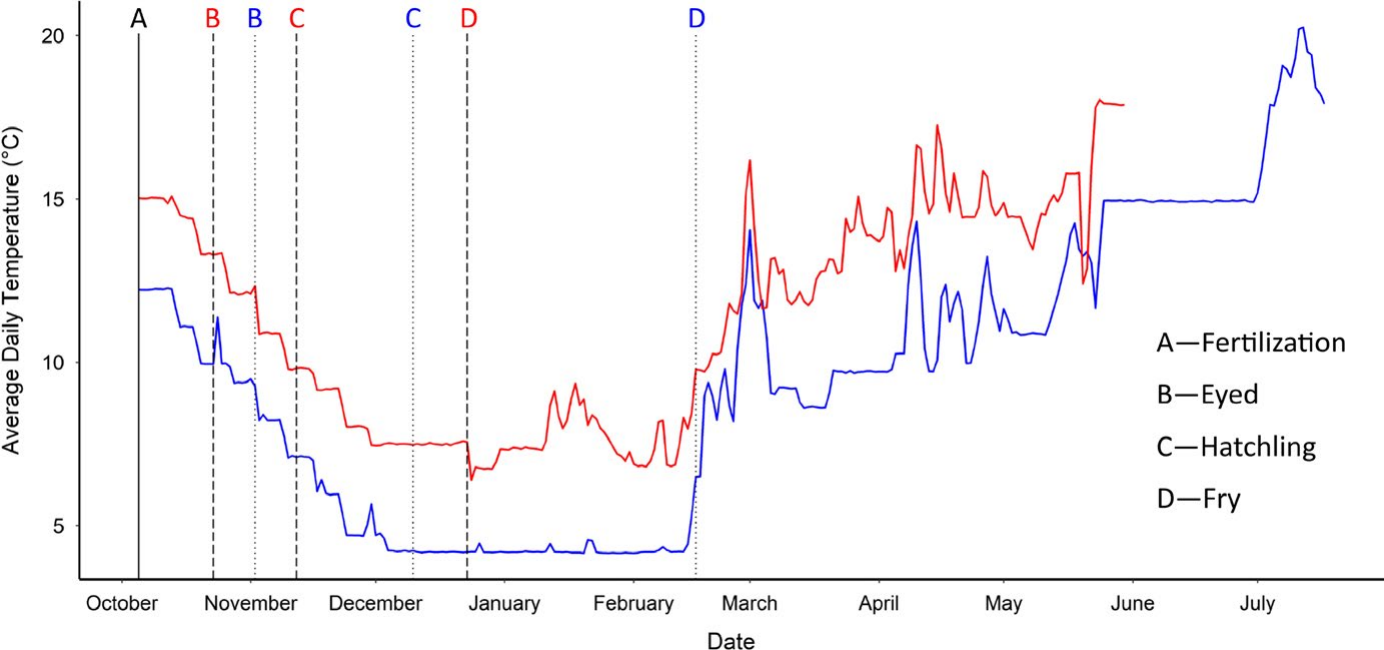
Recorded average daily temperature for each temperature regime in the rearing experimental design. Line and letter color represent rearing temperature regime: current—blue and elevated—red. Vertical lines represent sampling timepoints: solid—both current and elevated groups; dashed—elevated, dotted—current

At the aquatic facility, we filled individual containers with 90 g of eggs (~300 eggs) from each of the 15 females and added ~0.5 ml (4 drops) of pooled milt from the nine males. After gently swilling the egg–milt solution we added 60 ml of river water to activate the sperm (Lehnert, Helou, Pitcher, Heath, & Heath, 2018). After 2 min (when sperm should no longer be motile), we added river water mixed to 1,000 ng/ml concentration of cortisol (H4001; Sigma‐Aldrich Canada Co) dissolved in 90% ethanol (HPLC grade; Sigma‐Aldrich Canada Co) or 0 ng/ml control solution (ethanol and water only) for a 2‐hr incubation. These concentrations and exposure times were chosen as they have been shown in previous published studies to result in biologically relevant elevations of cortisol in the eggs (i.e., within 2 *SD* of control: Burton, Hoogenboom, Armstrong, Groothuis, & Metcalfe, 2011; Capelle, Semeniuk, Sopinka, Heath, & Love, 2017; Sopinka et al., 2017; Sopinka, Hinch, Healy, Raby, & Patterson, 2016). After the 2‐hr exposure, we rinsed the eggs using dechlorinated water, and removed three eggs from each cortisol replicate to verify the effectiveness of the cortisol manipulation. The remaining eggs were transferred to 4‐inch × 3‐inch incubation cells (each tray divided into 16 cells using metal dividers) within a vertical egg‐incubation stack that followed one of two temperature treatments. Eggs were incubated either at ambient water temperatures indicative of the “current” water temperature scenario or under the predicted “future climate change” scenario (i.e., elevated by 3°C based on predicted water temperature increases; Figure 2). The current temperature regime mirrored average water temperature seasonal patterns (2010–2014) recorded by a weather station close to the Credit River salmon spawning grounds as part of the Provincial Water Quality Monitoring Network (Site 06007605002; Government of Ontario). For the “future climate change” temperature regime, water temperatures were elevated by 3°C based on climate projections for the region (Vliet, Franssen, et al., 2013). This experimental design resulted in four treatment groups: current temperature—control, current temperature—cortisol‐dosed, elevated temperature—control, and elevated temperature—cortisol‐dosed. To control for maternal effects, each female's eggs were split across all four treatment combinations, where each treatment (egg cortisol treatment + temperature) had two replicate containers resulting in eight containers per female. Eggs from each container were then split across two replicate cells within each incubation stack, resulting in 16 incubation cells per female. This design allows for the control of container and position effects of rearing. Embryos were reared under 12:12 hr light: dark cycles. Daily water changes were completed, temperature was measured every 60 min (HOBO^®^ Water Temperature Pro v2 Data Logger; Onset) and dissolved oxygen (LabQuest 2: optical DO probe; Vernier) daily. Dissolved oxygen was consistently above 10 mg/L throughout incubation period.

### 2.3 Fertilization success, morphology, and survival

**Figure 3:**
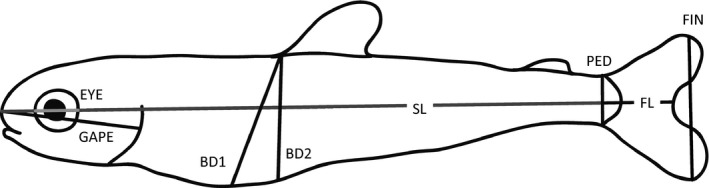
Morphological measurements of emerged juvenile Chinook salmon taken from photographs analyzed using ImageJ. Measurements include standard length (SL: gray), forked length (FL: gray and black), gape (GAPE), eye diameter (EYE: light gray), body depths 1 and 2 (BD1 & BD2), caudal peduncle width (PED), and caudal fin width (FIN)

Offspring development and mortality were assessed every two days. We removed and stored dead eggs in Stockard's solution (5% formaldehyde, 4% glacial acetic acid, 6% glycerin, 85% water) to determine fertilization success and at which developmental stage death occurred. Due to the effects of water temperature on development in this ectothermic species, offspring raised in the two temperature regimes reached development stages at distinct calendar days (Figure 2). We therefore equalized fish developmental stages based on Accumulated Thermal Units (ATUs), which is the sum of average daily temperatures, and is highly correlated to fish growth and development (Chezik, Lester, & Venturelli, 2014; Neuheimer & Taggart, 2007). Once offspring reached their emergence stage (complete absorption of the yolk sac: 835/830 ATUs on December 23, 2016/February 16, 2017, for elevated and current temperature, respectively), five offspring from each cell were haphazardly chosen and removed from the incubation stack, their body mass measured (±0.01 g), and an image taken for morphological analysis using ImageJ (https://imagej.nih.gov/ij/). From these images, we measured standard length (SL), forked tail length (FL), gape (GAPE: operculum flap to tip of nose), eye diameter (EYE), body depth one (BD1: perpendicular to standard length starting from dorsal fin) and body depth two (BD2: from dorsal fin to deepest part of the belly), caudal peduncle width (PED), and caudal fin width (FIN) to 0.01 mm (Figure 3).

### 2.4 Egg cortisol assay

To verify the success of the cortisol manipulation, we measured cortisol concentrations in both unfertilized and 2‐hr post‐treatment eggs. Briefly, we sampled three eggs from each treatment (unfertilized, control‐dosed, and cortisol‐dosed) for each of the 15 females (*N* = 45). We weighed, and blended eggs in 1.2 ml assay buffer, and then extracted the cortisol according to protocol as detailed in Capelle et al. (2017). Samples were stored in –80°C freezer until use in assay. Egg samples were run in triplicate using ELISA Cayman Cortisol kits in 1:57 dilution and were read at 412 nm on a plate reader. Intra‐ and interassay (i.e., plate) coefficients of variation were 8.1% and 20.4%, respectively.

### 2.5 Statistical analysis

We completed statistical analyses in R version 3.5.1 (R Core Team, 2018). We assessed model assumptions by graphical inspection: residuals versus fitted values were plotted to verify homogeneity, and the quantile–quantile plots of the residuals to verify normality. Since egg cortisol concentrations did not meet the normality assumption, we transformed egg cortisol concentrations data using Box‐Cox power transformation, *λ* = 0.242 (*MASS* package, Venables & Ripley, 2003). The effect of cortisol treatments on egg cortisol concentrations was analyzed using linear mixed model (LMM) with maternal identity as a random factor. We used the buildBinary function in *fullfact* package (Houde & Pitcher, 2016) to build the fertilization success and survival models, where each individual was assigned 1 when fertilized and 0 when unfertilized for fertilization success, or 1 when alive and 0 when dead for survival. Both models were tested using a binomial distribution generalized linear mixed model with maternal identity and incubation‐cell placement nested within incubation‐tray identity in the stack as random factors. The fertilization‐success model tested the cortisol treatment, temperature, and their interaction. The survival model included cortisol treatment, temperature, developmental stage, and all two‐way interactions.

The nine morphological measurements were incorporated into a principal components analysis (PCA; see Table 1 for means). We removed individuals with incomplete measurements due to image quality (*n*_removed_ = 94; *N*_analyzed_ = 1,006). Two components were significant under the Kaiser criterion (eigenvalue > 1) with Varimax orthogonal rotation. Loadings above 0.55 were considered significant—criteria were chosen to reduce significant loadings between components (Table 2). PC1 explained 32.7% of the variance in the model with positive significant loadings for mass, SL, FL, GAPE, and EYE explaining *structural size*. PC2 explained 30.6% of the variance with positive significant loadings for mass, BD1, and BD2 describing *body condition*. Cortisol treatment and rearing temperature effects on these two morphological components were determined using LMM with maternal identity, and cell placement nested in tray placement in the incubation stacks as random factors. The full model included fixed effects of cortisol treatment and rearing temperatures, as well as their interaction.

**Table 1:** Morphological traits (mean ± *SE*) of Chinook salmon fry across prenatal cortisol and water temperature treatments

Trait	Current temperature		Elevated temperature	
	Control	Cortisol‐dosed	Control	Cortisol‐dosed
Mass (g)	0.446 ± 0.003	0.443 ± 0.003	0.431 ± 0.004	0.434 ± 0.004
Standard length (mm)	33.8 ± 0.07	33.7 ± 0.07	33.2 ± 0.07	33.3 ± 0.07
Fork length (mm)	37.7 ± 0.08	37.6 ± 0.08	37.1 ± 0.08	37.1 ± 0.08
Gape (mm)	8.11 ± 0.02	8.12 ± 0.02	8.01 ± 0.02	8.05 ± 0.02
Eye width (mm)	2.20 ± 0.01	2.21 ± 0.01	2.17 ± 0.01	2.19 ± 0.01
Body depth 1 (mm)	6.26 ± 0.03	6.24 ± 0.03	6.18 ± 0.03	6.10 ± 0.04
Body depth 2 (mm)	7.76 ± 0.04	7.74 ± 0.04	7.87 ± 0.05	7.79 ± 0.05
Caudal peduncle (mm)	2.35 ± 0.01	2.33 ± 0.01	2.18 ± 0.01	2.21 ± 0.01
Caudal fin (mm)	7.46 ± 0.04	7.39 ± 0.04	7.35 ± 0.04	7.40 ± 0.04
Number of samples	238	273	253	242

**Table 2:** Principal component loadings for the morphology PCA. Loadings were considered significant >0.55

Trait	PC1 Structural size	PC2 Body condition
Mass	0.55	**0.61**
Standard length	**0.74**	0.49
Fork length	**0.76**	0.51
Gape	**0.71**	0.22
Eye width	**0.71**	−0.14
Body depth 1	0.21	**0.87**
Body depth 2	0	**0.86**
Caudal peduncle	0.54	0.22
Caudal fin	0.40	0.53
Percent variance explained	32.7%	30.6%
Eigenvalue	4.5	1.2

Significant Loadings (>0.55) are in bold.

For all models, we tested fixed effects using likelihood ratios (LRT) by fitting full models for fertilization success and survival with maximum likelihood estimation (Zuur, Ieno, Walker, Saveliev, & Smith, 2009). If all interactions were nonsignificant (*p* > .05), interactions were removed from the model and main effects were tested sequentially and compared using LRT with maximum likelihood (ML) estimation. When a significant interaction was present (*p* ≤ .05), all interactions were retained and refitted using restricted maximum likelihood estimation (REML), and pairwise comparisons using Tukey's HSD in *emmeans* package (Lenth, Singmann, Love, Buerkner, & Herve, 2018). If no significant interactions were present, but a main effect was, the model was refitted using REML and tested using Tukey's HSD pairwise comparisons.

## 3 RESULTS

### 3.1 Egg cortisol

**Figure 4:**
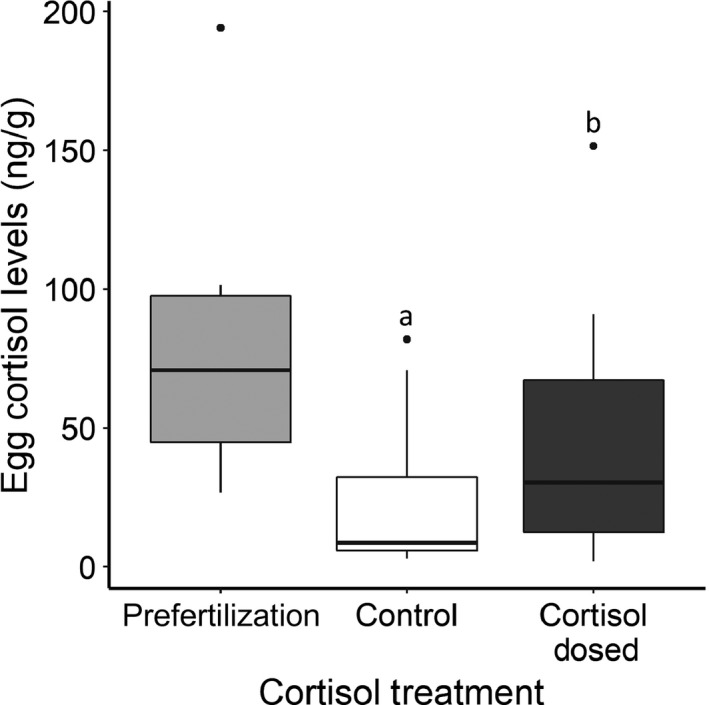
Cortisol concentrations in the prefertilized eggs and in manipulated eggs after the 2‐hr cortisol treatment incubation: control 0 ng/ml or cortisol‐dosed 1,000 ng/ml. Cortisol‐dosed eggs had significantly higher cortisol levels than control‐treated eggs

Cortisol concentrations were significantly higher in the cortisol‐treated than control‐treated eggs (LMM: *t* = 6.9, *p* < .001, *n*_cort_ = 15, *n*_control_ = 15; Figure 4). The cortisol treatment mean (mean ± *SD*, range; 75.2 ± 42.4, 26.6–194.1 ng/g) was within two standard deviation of the control‐treated eggs (22.8 ± 25.4, 2.9–81.9 ng/g) confirming a biologically relevant cortisol elevation (i.e., within 1–2 *SD* and thus not a pharmacological treatment; Burton et al., 2011; Sopinka et al., 2016). The prefertilized nonmanipulated eggs were within one standard deviation of both cortisol‐dosed and control eggs (43.8 ± 42.6, 1.9–151.5 ng/g).

### 3.2 Fertilization success and survival

**Figure 5:**
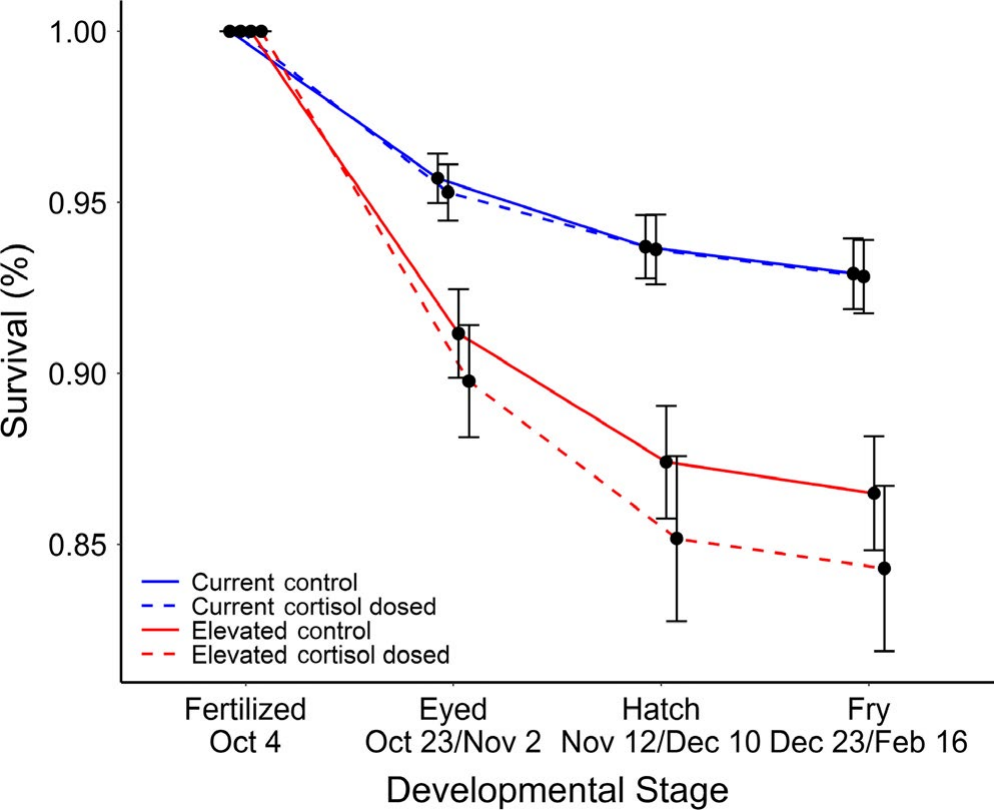
Percent survival across developmental stages. Error bars denote *SE*. At hatch, elevated‐reared fish had lower survival than current temperature fish and at eyed, cortisol‐dosed fish reared in elevated temperatures had significantly lower survival than cortisol‐dosed fish reared in current temperatures

There was no interactive effect of temperature and cortisol exposure on fertilization success (LRT: *χ* ^2^ = 0.094, *p* = .76). Upon removing the interaction, neither temperature (*χ* ^2^ = 0.39, *p* = .53) nor cortisol treatment (*χ* ^2^ = 0.16, *p* = .69) was significant. Similarly, there was no temperature by cortisol treatment interaction on survival (*χ* ^2^ = 1.69, *p* = .19), meaning cortisol‐dosed offspring did not have a higher survival than control offspring within temperatures. However, there was a significant temperature by stage interaction on survival (LRT: *χ* ^2^ = 10.51, *p* = .0052; Figure 5). At eyed stage, cortisol‐dosed offspring raised in elevated temperatures had a lower survival rate than those incubated in current temperatures (Tukey's HSD: current—cortisol‐dosed & control vs. Elevated—cortisol‐dosed: *p* < .001). Control offspring reared at elevated temperatures had a marginally lower survival than current—control reared offspring, and no statistical difference than current‐cortisol offspring (Tukey's HSD: current—control, current—cortisol‐dosed vs. elevated—control: *p* = .055, *p* = .10). At the hatch stage, offspring raised in elevated temperatures had a lower survival rate than those raised in current temperatures (Tukey's HSD: all combinations of current—cortisol‐dosed and control vs. elevated—cortisol‐dosed and control: *p* < .05). At the fry stage, there were no statistical differences between fish raised in elevated versus current temperatures (Tukey's HSD: all combinations of current—cortisol‐dosed and control vs. elevated—cortisol‐dosed and control: *p* > .10). The cortisol treatment and stage interaction had a marginal effect on survival (*χ* ^2^ = 4.66, *p* = .097), however the specifics of this effect were not detected in post hoc analysis.

### 3.3 Structural size and body condition

**Figure 6:**
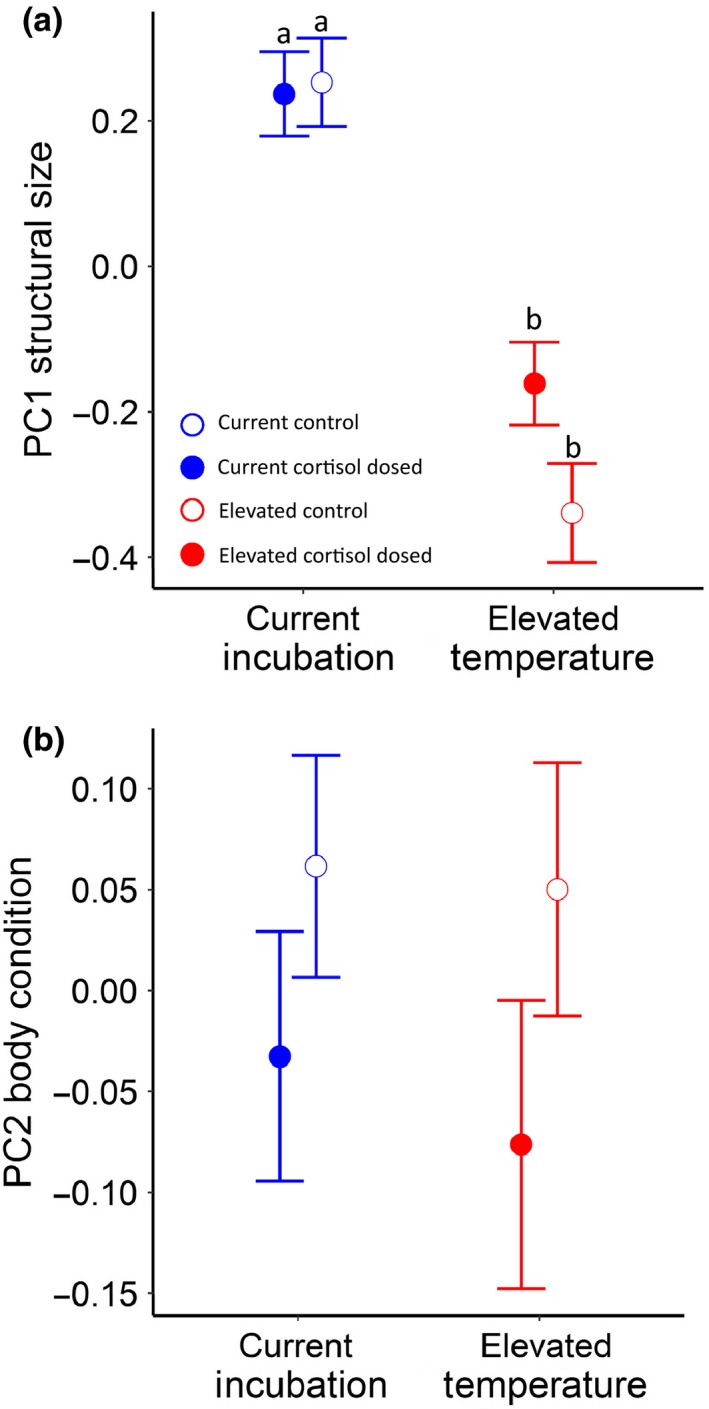
Morphology PCA scores for (a) PC1—structural size and (b) PC2—body condition. (a) Offspring raised in elevated temperatures were structurally smaller than those raised in current. (b) No differences in body condition across the treatment groups. Error bars denote *SE*

There was no temperature by cortisol treatment interaction on fry structural size (i.e., morphology PC1, *χ* ^2^ = 2.00, *p* = .16), with a strong temperature effect on structural size (*χ* ^2^ = 8.76, *p* = .003; Figure 6a), where fry raised in elevated temperatures were significantly smaller than those in current temperatures. Cortisol treatment marginally affected fry body size (*χ* ^2^ = 3.72, *p* = .054), where cortisol‐dosed fry were larger than control‐dosed. There was no interaction between temperature and cortisol treatment on body condition (PC2, *χ* ^2^ = 0.13, *p* = .72), and following the removal of the interaction there were no temperature or cortisol effects on body condition (temperature: *χ* ^2^ = 0.003, *p* = .99; cortisol: *χ* ^2^ = 0.33, *p* = .57; Figure 6b).

## 4 DISCUSSION

Elevated temperatures during early development can have diverse “negative” phenotypic, performance, and fitness‐related effects on aquatic organisms. Based on predictions from the environmental matching hypothesis, we aimed to test whether prenatal exposure to elevated egg cortisol buffers these temperature effects. Our results confirm the effects of warmer water temperatures, but suggest that exposure to elevated egg cortisol does little to ameliorate these impacts contrary to predictions of the environmental match hypothesis (Sheriff & Love, 2013).

### 4.1 Early survival in warming waters

Warmer temperatures, but not early cortisol exposure, affected offspring survival with fish raised in elevated temperatures having lower survival at the eyed and hatchling stages. Elevated incubation temperatures above species' preferred temperature range result in lower survival in juvenile salmonids, regardless of whether the elevation follows a stable temperature increase (Tang, Bryant, & Brannon, 1987; Whitney et al., 2013) or an oscillating temperature regime (Geist et al., 2006; Taranger & Hansen, 1993). Since salmon are ectothermic, offspring are sensitive to temperature increases, especially during early development (Beacham & Murray, 1990; Tang et al., 1987) when offspring respond strongly to environmental cues (Monaghan, 2008). This associated decline in embryo survival has been attributed to temperature‐dependent increases in yolk coagulation (McCollough, 1999) and increases in developmental deformities (Cingi, Keinänen, & Vuorinen, 2010; Fraser, Hansen, Fleming, & Fjelldal, 2015).

Contrary to our prediction, exposure to elevated egg cortisol (mimicking maternal stress) did not dampen the negative effect of elevated incubation temperatures on offspring survival, and in one instance (i.e., eyed stage) may have resulted in lower survival. Previous studies have found a diversity of effects of maternal stress on offspring survival in fish (Sopinka et al., 2017). In Atlantic salmon (*Salmo salar*) where mothers received cortisol injections and eggs were incubated under mild hyperthermia (+2°C), there were no effects on offspring survival (Eriksen, Bakken, Espmark, Braastad, & Salte, 2006; Eriksen, Espmark, Braastad, Salte, & Bakken, 2007). However, although under benign environments some studies have reported either lower (*Oncorhynchus mykiss*: Li, Bureau, King, & Leatherland, 2010) and even higher (*O. tshawytscha*: Capelle et al., 2017) early survival for offspring exposed to elevated egg cortisol, the majority of studies have reported no effect across a diversity of fish species (*Danio rerio*: Nesan & Vijayan, 2016; *Salmo trutta*: Sloman, 2010; *O. kisutch*, *O. keta*,* O. nerka*: Sopinka et al., 2016; Sopinka, Hinch, Healy, Harrison, & Patterson, 2015; *O. kisutch*: Stratholt, Donaldson, & Liley, 1997). Considering these contrasting results both within and across fish species, it may not be surprising that exposure to elevated egg cortisol was incapable of ameliorating survival of elevated water temperatures. However, studies should continue to explore the role of maternally derived cortisol, as the dose used in this study may not have matched the severity of environmental stressor (i.e., dose not representative of maternal environmental stress). Future studies could work to determine the exact cortisol dose that matches a given environmental stressor by taking a dose‐response approach. Additionally, it is possible that the prenatal cortisol dose used in this study may have downstream (indirect) impacts on survival at later developmental stages through changes in performance (e.g., morphology, physiology, and behavior), especially when under the effects of a stressful environment. These effects largely remain to be studied in fish within a “stressful” environmental context (although see Capelle, 2017).

Survival from egg to exogenously feeding fry represents an important bottleneck that limits offspring migration success to the lake or ocean (Groot & Margolis, 1991). Thus, elevated temperatures in the freshwater riverine stage may have long‐lasting effects on population demography. In our study, effects of temperature on survival were significant during early stages of development—the eyed and hatching stage. As such, if managers were only able to examine survival at the fry stage, temperature effects would not be detected, meaning that conservation measures could be delayed while populations are still declining. Therefore, quantifying relative survival across freshwater life stages to determine which are at higher risk of mortality due to stressors such as temperature is important, especially since many Chinook salmon populations in their native range are in decline (Kope et al., 2016).

### 4.2 Temperature and prenatal cortisol effects on fry structural size and body condition

Morphology, and in particular, body size, impacts juvenile performance metrics such as foraging (Johnsson, 1993), or predator avoidance (Tucker, Hipfner, & Trudel, 2016) that ultimately contribute to variation in fitness. We found that fry raised in elevated temperatures were smaller than under current water conditions, with no apparent temperature effects on body condition. Previous studies indicate that elevated water temperatures generate smaller body size in fish larva and fry (Beacham & Murray, 1990; Murray & Mcphail, 1988; Whitney et al., 2014), presumably via the higher metabolic costs of living in warmer waters (Kingsolver & Huey, 2008; Sheridan & Bickford, 2011) since fish may allocate less energy for growth and nonmaintenance activities to compensate for the thermal stress. Interestingly, biothermal modeling in ectotherms has predicted selection for smaller body size, and maturation at a younger age (and smaller body size: Daufresne et al., 2009). As such, smaller body size per se may not in itself be inherently negative, especially when body condition was unaffected, suggesting smaller fish are not energetically limited. Additionally, changes in phenotype do not necessarily translate into changes in performance, and most importantly, fitness.

We did not detect any significant effects of exposure to elevated prenatal cortisol on either structural size (PC1) or body condition (PC2) within elevated rearing temperatures. Thus, our results do not support predictions from the environmental matching hypothesis that early developmental exposure to elevated cortisol better match offspring to warmer waters through changes in body size or condition (again, assuming as outlined above that smaller body size is a “negative” trait response to elevated water temperature). As with our observed effects for survival, elevated temperatures may have an overwhelming impact (and possibly even adaptive) as an environmental effect on growth and development compared to our modulatory maternal effect, prenatal egg cortisol. The marginal effect of prenatal cortisol on overall body condition (regardless of temperature treatment) suggests that prenatal cortisol still has the potential to enact phenotypic change. Previous work has shown that exposure to elevated prenatal cortisol often results in decreased body size in benign environments (Sopinka et al., 2017), but few studies have examined the interactive effects of prenatal cortisol and stressful environments on body size. Atlantic salmon fry whose mothers were exposed to cortisol close to spawning had smaller body length and body mass than control fry when incubated in (+2°C) elevated temperatures at fry stage (Eriksen et al., 2006). Likewise, Chinook salmon exposed to a similar dose of egg cortisol (1,000 ng/ml) as the current study and raised in lower quality water conditions were smaller (Capelle, 2017). However, under a lower dose of egg cortisol (300 ng/ml) and under the same low‐quality water conditions fish were larger than higher dose fish suggesting a match between the degree of the prenatal signal and the relative quality of the postnatal environment (Capelle, 2017). These complex results highlight the importance of following offspring phenotype across life stages, as maternal stress may induce delayed effects on phenotype and performance. Importantly, it must be emphasized that despite having lower survival, smaller offspring living in stressful environments may benefit via less caloric intake needed (Gillooly, Brown, & West, 2001), or better escape performance (Chin et al., 2009).

### 4.3 Early life in stressful environments

Wild fish populations are globally in decline (Dulvy, Jennings, Rogers, & Maxwell, 2006), and a number of salmonid species, including Chinook, are currently at population extinction risk, presumably due to the direct and indirect effects of climate change (Crozier, 2015). Early development in juvenile salmon occurs in a dynamic environment; streams and rivers can change considerably in temperatures both daily and seasonally (Caissie, 2006). Due to this fluctuating tendency and higher surface area to volume, streams and rivers are more likely to be affected by climate change through temperature increases and extreme changes in flow (Hill, Hawkins, & Jin, 2014; van Vliet, Ludwig, & Kabat, 2013). Since environmental variation during development plays a large role in generating variability in offspring phenotypes through developmental plasticity and flexibility, it is important to determine whether juvenile salmon have the capacity to respond to the rapid rate of climate change in their environments. In our study, we examined the role of a stress‐induced maternal effect, egg cortisol, as a potential modulator of offspring phenotype and performance in response to elevated water temperatures induced by anthropogenic change. More broadly, we were also able to investigate predictions of the environmental matching framework to test whether maternal signals may modulate offspring responses adaptively in response to future stressful environments. Although we did not find support that elevated egg cortisol led to altered offspring phenotypes or survival during early freshwater stages, it is still possible that exposure to maternal stress modulates phenotypes or performance at later important developmental stages. Further work examining a range of cortisol doses on offspring phenotype and performance under matched environmental conditions is warranted. Additionally, interpreting the fitness impacts of maternal stress within an environmental context continues to be highly important for determining how maternal effects may assist species' responses to rapid environmental changes.

## CONFLICT OF INTEREST

Authors have no conflicts of interest.

## AUTHOR CONTRIBUTIONS

TRW, CAD, and OPL conceived ideas, and designed methodology; TEP provided rearing facilities; all authors contributed to data collection; TRW, CAD, and OPL ran analyses and led writing of the manuscript with input from all authors.

## Data Availability

Data have been archived in the Dryad repository https://doi.org/10.5061/dryad.2rbnzs7j7.
